# How does severe acute respiratory syndrome coronavirus 2 (SARS-CoV-2) achieve immune evasion?: A narrative review

**DOI:** 10.1097/MD.0000000000037780

**Published:** 2024-04-19

**Authors:** Yahu Bai, Kang Ning

**Affiliations:** a Department of Respiratory, The First Affiliated Hospital of Shandong First Medical University, Shandong Provincial Qianfoshan Hospital, Shandong Institute of Respiratory Diseases, Shandong Institute of Anesthesia and Respiratory Critical Medicine, Jinan, China; b Department of Respiratory, Occupational Diseases Hospital of Shandong First Medical University, Shandong Province Hospital of Occupational Diseases, Jinan, China.

**Keywords:** immune evasion, liquid–liquid phase separation, nucleocapsid protein, SARS-CoV-2, virus

## Abstract

COVID-19 caused by the novel coronavirus, severe acute respiratory syndrome coronavirus 2, (SARS-CoV-2) is a highly contagious disease known for its significant lung damage. Although the impact of the COVID-19 pandemic on our daily lives has been limited, the virus has not vanished entirely and continues to undergo mutations. This calls for a concentrated focus on the matter of SARS-CoV-2 immune evasion. Drawing on observations of immune escape mechanisms in other viruses, some scholars have proposed that liquid–liquid phase separation might play a crucial role in SARS-CoV-2’s ability to evade the immune system. Within the structure of SARS-CoV-2, the nucleocapsid protein plays a pivotal role in RNA replication and transcription. Concurrently, this protein can engage in phase separation with RNA. A thorough examination of the phase separation related to the nucleocapsid protein may unveil the mechanism by which SARS-CoV-2 accomplishes immune evasion. Moreover, this analysis may provide valuable insights for future development of innovative antiviral drugs or vaccines.

## 1. Introduction

Since the outbreak of the COVID-19 pandemic in December 2019, the novel coronavirus, severe acute respiratory syndrome coronavirus 2 (SARS-CoV-2), has spread worldwide, with the cumulative number of confirmed cases exceeding 770 million and the cumulative death toll surpassing 6.9 million.^[1]^ Due to SARS-CoV-2’s high transmissibility and severe pathogenicity, it has caused a severe global public health crisis. On May 5, 2023, the World Health Organization declared that the COVID-19 pandemic no longer constituted a “public health emergency of international concern.” However, this does not mean the end of COVID-19 as a global health threat. Even as populations in various countries have achieved high levels of immunity through natural infection and/or vaccination, sporadic cases continue to be reported, and “breakthrough” infections are not uncommon, suggesting that SARS-CoV-2 may achieve immune escape through its structural characteristics. Understanding “how the virus achieves immune escape” has been a focal point of research for experts worldwide. Combining the structural features of SARS-CoV-2 and findings from previous research on SARS-CoV, some scholars have proposed that liquid–liquid phase separation (LLPS) may be an important mechanism by which SARS-CoV-2 accomplishes immune evasion. This review searched PubMed, Medline, and Web of Science using the keywords “SARS-CoV-2,” “Nucleocapsid protein,” “Liquid–liquid phase separation,” and “Immune evasion.” Through systematic organization of relevant clinical studies, basic research and reviews, it provides a comprehensive exploration of the principles of LLPS, the structure of SARS-CoV-2, and the repercussions of LLPS involving the SARS-CoV-2 nucleocapsid protein on the human immune system. It provides an in-depth exploration of the potential for SARS-CoV-2 nucleocapsid protein to achieve immune evasion through LLPS, with the aim of offering valuable insights for the development of new antiviral drugs or vaccines.

## 2. Liquid–liquid phase separation (LLPS)

LLPS, commonly referred to as phase separation, was initially used in physical chemistry to describe the phenomenon of 2 mixed liquid phases spontaneously separating from each other. In theory, when specific large molecules uniformly dispersed in a solution reach a critical concentration threshold, phase separation occurs spontaneously.^[2]^ The most common example of LLPS in everyday life is when oil droplets are added to water; the oil droplets do not blend with the water but instead float on the water’s surface. This phenomenon is primarily driven by multivalent weak interactions,^[3]^ and whether phase separation occurs depends to a large extent on the concentration and characteristics of the large molecules and the solution, as well as environmental conditions, including temperature, the type and concentration of salt solutions, and pH, among others.^[4]^

The phenomenon of phase separation is not only present in physical chemistry but also within biological cells. In 2009, Brangwynne et al^[5]^ discovered that P granules in the primitive reproductive cells of roundworms could flow and fuse like liquid droplets, providing crucial insight into the study of phase separation within cells. Furthermore, phase separation has allowed us to glimpse the complexity of biology. As it is well known, to carry out a range of different cellular functions within limited space, cells contain various membraneless organelles (MLOs) such as Cajal bodies, stress granules, and spindle apparatus, among others.^[6,7]^ However, how these MLOs achieve compartmentalization and fulfill their cellular functions has long fascinated many researchers. Increasing evidence now suggests that phase separation forms the foundation for the formation of MLOs within cells. Although the precise criteria for defining phase separation are still evolving, a consensus has been reached in this field that the following 3 criteria should be met for the formation of MLOs through phase separation: maintaining a spherical shape, fusing upon contact, and exchanging molecules with the surrounding environment.^[8]^ Consequently, through phase separation, specific molecules aggregate within the cell, bringing a degree of order to the “chaotic” cellular environment. Similarly, it is through phase separation that MLOs can exchange substances with their surrounding environment.

A significant amount of research has confirmed that phase separation can drive the formation of condensates for proteins, RNA, and other biological macromolecules,^[9]^ subsequently participating in a series of cellular activities. These cellular activities include cell division, signal transduction, higher-order genome organization, gene regulation, the formation of presynaptic and postsynaptic dense signal components, and protein autophagic degradation.^[10]^ Aberrations in phase separation can lead to various neurodegenerative diseases such as amyotrophic lateral sclerosis,^[11,12]^ frontotemporal dementia,^[13]^ and Alzheimer’s disease.^[14]^ Additionally, various cancers, such as prostate cancer, breast cancer, and liver cancer, have also been confirmed to be associated with abnormalities in phase separation. Meanwhile, phase separation not only plays a significant role in eukaryotes but also serves an indispensable function in bacteria. Within bacteria, phase separation can mediate the formation of subcellular structures like BR-bodies (bacterial ribonucleoprotein bodies), RNA polymerase clusters, single-stranded DNA-binding protein, division proteins like FtsZ and SImA, and more.

Similarly, the phenomenon of phase separation of viral components has been confirmed in various viruses, such as HIV-1^[15]^ and HSV-1.^[16]^ Li et al^[7]^ elucidated the roles of phase separation in virus entry and uncoating, viral nucleic acid synthesis and replication, virus assembly, virus release, and immune evasion in their article. Furthermore, virus protein-driven phase separation serves 2 functions. One is the formation of the so-called “virus factory” or “virus inclusion body.” The “virus factory” is the site for virus replication and assembly, as well as the concentration of specific proteins and nucleic acids. The other function is the formation of molecular condensates, which are not the primary sites for virus RNA synthesis but are related to the assembly and transport of virus components (i.e., proteins and nucleic acids). When the body is invaded by a pathogen, the innate immune system responds rapidly, initiating a cascade of signaling events through the recognition and activation of pattern recognition receptors, leading to the activation of cellular defense mechanisms. To evade the host cell’s defenses, viruses interfere with host cells through phase separation. According to research, phase separation-mediated interference of viruses with host cells mainly involves 2 mechanisms: one is based on the direct interaction between virus condensates and specific host genes, and the other is based on interactions with cellular proteins, isolating key cellular proteins such as proteins that trigger the innate immune response in the cell.^[17]^ Today, more and more research has found that the components of SARS-CoV-2 also undergo phase separation, and phase separation may be an important mechanism for SARS-CoV-2 to achieve immune evasion. Therefore, in-depth research and exploration of SARS-CoV-2 and phase separation may provide strategies and insights for the development of new antiviral drugs or vaccines.

## 3. SARS-CoV-2 and liquid–liquid phase separation (LLPS)

### 3.1. Overview of SARS-CoV-2 structure

SARS-CoV-2 is a novel β-coronavirus with approximately 79% genetic homology to the SARS-CoV genome and about 50% genetic homology to MERS-CoV.^[18,19]^ SARS-CoV-2 is a single-stranded, positive-sense RNA virus with a genome length of approximately 29.9 kb.^[20]^ It consists of 14 open-reading frames that encode 16 nonstructural proteins, 4 structural proteins, and 9 accessory proteins. The 16 nonstructural proteins are encoded by ORF1a and ORF1b and possess different enzymatic functions, playing a role in regulating virus RNA replication and transcription.^[21]^ The 4 structural proteins are the spike protein (S), membrane protein (M), envelope protein (E), and nucleocapsid protein (N), which are essential for virus assembly. Among these, the S protein is a surface-anchored viral glycoprotein composed of 2 noncovalently bound subunits, S1 and S2. The S1 subunit is responsible for binding to host cell receptors, while the S2 subunit anchors the virus to the cell membrane and facilitates fusion between the virus and the cell membrane.^[22,23]^ The M protein is the most abundant structural protein in SARS-CoV-2, determining the shape of the viral envelope. It interacts with other structural proteins to facilitate virus particle assembly: M protein binding with N protein stabilizes the viral nucleocapsid (N protein–RNA complex) and the internal core of the virus particle, while its interaction with the E protein helps maintain the shape of the virus particle and promotes release.^[24]^ The E protein is the smallest structural protein in SARS-CoV-2 and can form a pentameric ion channel. It plays a role in the assembly and budding of coronaviruses.^[25,26]^ The 9 accessory proteins include ORF3a, ORF3b, ORF6, ORF7a, ORF7b, ORF8, ORF9b, ORF9c, and ORF10.^[21]^ These accessory proteins are not essential for coronavirus replication and transcription but can aid the virus in adapting to the host and evading the human immune system.^[27]^

### 3.2. SARS-CoV-2 nucleocapsid protein

The SARS-CoV-2 N protein, as a core component of the viral particle, is a 46 kDa multi-domain RNA-binding protein. It includes 2 known structural domains, the N-terminal domain and the C-terminal domain (CTD), along with 3 predicted intrinsically disordered regions.^[28]^ Previous research has established that its N-terminal domain is an RNA-binding domain that can interact with various RNA and DNA sequences, both specific and nonspecific. Additionally, the CTD is known to form higher-order structures through dimerization/oligomerization. Recent studies have found that the CTD is a hidden domain capable of binding ATP and nucleic acids.^[29]^ A detailed understanding of the N protein’s structure is crucial for uncovering its biological functions and providing a foundation for the development of new drugs. Currently, most research suggests that the primary role of the N protein is to bind to the viral genomic RNA, encapsulating it into ribonucleoprotein (RNP) complexes, which are essential for maintaining the ordered conformation of RNA during replication and transcription. Furthermore, the N protein has other functions, including hijacking host cells for RNA replication and transcription, nucleocapsid assembly, and immune regulation.^[30]^ Moreover, the N protein is highly immunogenic and serves as an antigen.^[31]^ It is a determinant of virulence and the disease mechanism, with its expression significantly increasing upon entry into host cells, leading to the induction of humoral and cellular immune responses.^[32,33]^ Therefore, many researchers consider the N protein a critical target for clinical rapid diagnostics and vaccine development.

### 3.3. SARS-CoV-2 N protein and liquid–liquid phase separation (LLPS)

Shortly after the emergence of SARS-CoV-2, Cascarina and Ross^[34]^ and Chen et al^[35]^ based on the structural characteristics and their research on the SARS-CoV N protein, predicted that the SARS-CoV-2 N protein may undergo phase separation with viral genomic RNA in the body and participate in the assembly of viral particles. Subsequently, Zhao et al^[36]^ utilized recombinant N protein tagged with mEGFP. They confirmed its capability to undergo phase separation from RNA through electrophoretic mobility shift assay and observed the dynamic process of phase separation between N protein and RNA using time-lapse microscopy. As research has advanced, it has become increasingly clear that the phase separation of the N protein is induced by RNA and regulated by phosphorylation of the SR domain.^[37,38]^ Furthermore, the N protein not only drives phase separation but also divides host cell proteins (such as hnRNPA2 and FUS) into liquid phases, thereby recruiting the necessary components to facilitate virus replication.^[17]^ Therefore, a deeper understanding of the N protein and the principles of N protein phase separation can not only reveal the interactions between the host and the virus but also provide new insights for the development of antiviral strategies or novel drug therapies.

Based on the structure of the N protein and the principles of N protein phase separation, different scholars have proposed various antiviral strategies. Wang et al^[39]^ discovered that the dimerization domain of the N protein can inhibit the innate immune system’s antiviral response by suppressing Lys63-linked polyubiquitination and mitochondrial antiviral-signaling aggregation. Therefore, targeting the N protein dimerization domain with peptides can disrupt phase separation and inhibit the replication of SARS-CoV-2 in the body. Iserman et al^[40]^ found that N protein phase separation is driven by specific RNA sequences and structures, suggesting that these specific RNA sequences and structures could potentially be used directly in the development of antiviral therapies. Because phosphorylation of the SR region of the N protein can weaken RNA-induced phase separation and viral RNA transcription, Savastano et al^[41]^ proposed that activators of SR protein kinase 1 could be potential antiviral drugs. The latest research reports that the N protein can achieve inhibition of stress granule formation by undergoing phase separation with stress granule proteins G3BP1/2 through its N-terminal IDR. Therefore, small molecules disrupting the interaction between N protein and stress granule-related proteins may serve as a direction for developing new antiviral drugs.^[7]^ Dang et al^[42]^ believe that small molecules regulating the interaction between the N protein and nucleic acids are crucial for intervening in the virus lifecycle, and thus, these molecules hold promise for the development of novel antiviral drugs. Furthermore, targeting the impact of the N protein on the immune system may provide insights into antiviral therapy. Chau et al^[38]^ suggest that N protein phase separation can disrupt the liquid droplets of the mitochondrial antiviral-signaling, which in turn induces the activation of interferon-1 by IRF3 and nuclear factor κB in the body, leading to the suppression of the innate immune response.^[39]^ In summary, the N protein and its phase separation play important roles in virus assembly, replication, and the response to the host cell’s immune system. Disrupting N protein phase separation theoretically can inhibit virus assembly and replication, making drugs targeting N protein phase separation potentially valuable in the development of new antiviral drugs.

## 4. Concluding remarks and future perspectives

For centuries, influenza virus pandemics have been a part of human history, with the 1918 “Spanish flu” being the most severe influenza pandemic in recent history. Although the COVID-19 pandemic that lasted for over 3 years has ended the global state of emergency, the presence of the novel coronavirus and its potential for variations still brings about uncertainties. Therefore, the enhancement of preventive and diagnostic capabilities, as well as the ability to respond to localized infections and outbreaks, remains crucial. Given the possibility of immune escape by SARS-CoV-2 variants, a deeper understanding of the structure of SARS-CoV-2 and the separation of the N protein may contribute to the prevention and treatment of COVID-19. Furthermore, to unravel the molecular mechanisms behind the immune escape achieved by the separation of the N protein and to develop novel antiviral drugs or vaccines, collaborative efforts across medical, biological, and chemical fields are essential, crossing departments, disciplines, and borders. It is believed that the management of this pandemic can provide an opportunity for a deeper insight into the immune escape mechanisms of the virus through the collective efforts of all.

## Author contributions

Writing—original draft: Yahu Bai.

Writing—review and editing: Kang Ning.

## References

[1] Geneva:World Health Organization. *WHO Coronavirus Disease (COVID-19) Dashboard*. [EB/OL]. Available at: https://covid19.who.int/table Accessed October 23, 2023.

[2] Zhang JZ Mehta S Zhang J. Liquid–liquid phase separation: a principal organizer of the cell’s biochemical activity architecture. Trends Pharmacol Sci. 2021;42:845–56. 34373114 10.1016/j.tips.2021.07.003 PMC8858030

[3] Li Q Wang X Dou Z. Protein databases related to liquid–liquid phase separation. Int J Mol Sci. 2020;21:6796. 32947964 10.3390/ijms21186796 PMC7555049

[4] Alberti S Gladfelter A Mittag T. Considerations and challenges in studying liquid–liquid phase separation and biomolecular condensates. Cell. 2019;176:419–34. 30682370 10.1016/j.cell.2018.12.035 PMC6445271

[5] Brangwynne CP Eckmann CR Courson DS. Germline P granules are liquid droplets that localize by controlled dissolution/condensation. Science. 2009;324:1729–32. 19460965 10.1126/science.1172046

[6] Zhao YG Zhang H. Phase separation in membrane biology: the interplay between membrane-bound organelles and membraneless condensates. Dev Cell. 2020;55:30–44. 32726575 10.1016/j.devcel.2020.06.033

[7] Li H Ernst C Kolonko-Adamska M. Phase separation in viral infections. Trends Microbiol. 2022;30:1217–31. 35902318 10.1016/j.tim.2022.06.005

[8] Su J Wilson M Samuel C. Formation and function of liquid-like viral factories in negative-sense single-stranded RNA virus infections. Viruses. 2021;13:126. 33477448 10.3390/v13010126 PMC7835873

[9] Uversky VN. Intrinsically disordered proteins in overcrowded milieu: membrane-less organelles, phase separation, and intrinsic disorder. Curr Opin Struct Biol. 2017;44:18–30. 27838525 10.1016/j.sbi.2016.10.015

[10] Zhang H Ji X Li P. Liquid-liquid phase separation in biology: mechanisms, physiological functions and human diseases. Sci China Life Sci. 2020;63:953–85. 32548680 10.1007/s11427-020-1702-x

[11] Gui X Luo F Li Y. Structural basis for reversible amyloids of hnRNPA1 elucidates their role in stress granule assembly. Nat Commun. 2019;10:2006. 31043593 10.1038/s41467-019-09902-7 PMC6494871

[12] Patel A Lee HO Jawerth L. A liquid-to-solid phase transition of the ALS protein FUS accelerated by disease mutation. Cell. 2015;162:1066–77. 26317470 10.1016/j.cell.2015.07.047

[13] Mann JR Gleixner AM Mauna JC. RNA binding antagonizes neurotoxic phase transitions of TDP-43. Neuron. 2019;102:321–38.e8. 30826182 10.1016/j.neuron.2019.01.048 PMC6472983

[14] Kostylev MA Tuttle MD Lee S. Liquid and hydrogel phases of PrPC linked to conformation shifts and triggered by Alzheimer’s Amyloid-β Oligomers. Mol Cell. 2018;72:426–43.e12. 30401430 10.1016/j.molcel.2018.10.009 PMC6226277

[15] Monette A Niu M Chen L. Pan-retroviral nucleocapsid-mediated phase separation regulates genomic RNA positioning and trafficking. Cell Rep. 2020;31:107520. 32320662 10.1016/j.celrep.2020.03.084 PMC8965748

[16] Metrick CM Koenigsberg AL Heldwein EE. Conserved outer tegument component UL11 from Herpes Simplex Virus 1 is an intrinsically disordered, RNA-binding protein. mBio. 2020;11:e00810–20. 32371601 10.1128/mBio.00810-20 PMC7403781

[17] Brocca S Grandori R Longhi S. Liquid–liquid phase separation by intrinsically disordered protein regions of viruses: roles in viral life cycle and control of virus–host interactions. Int J Mol Sci. 2020;21:9045. 33260713 10.3390/ijms21239045 PMC7730420

[18] Hu B Guo H Zhou P. Characteristics of SARS-CoV-2 and COVID-19. Nat Rev Microbiol. 2021;19:141–54. 33024307 10.1038/s41579-020-00459-7 PMC7537588

[19] Lu R Zhao X Li J. Genomic characterisation and epidemiology of 2019 novel coronavirus: implications for virus origins and receptor binding. Lancet. 2020;395:565–74. 32007145 10.1016/S0140-6736(20)30251-8 PMC7159086

[20] Wang MY Zhao R Gao LJ. SARS-CoV-2: structure, biology, and structure-based therapeutics development. Front Cell Infect Microbiol. 2020;10:587269. 33324574 10.3389/fcimb.2020.587269 PMC7723891

[21] Bai C Zhong Q Gao GF. Overview of SARS-CoV-2 genome-encoded proteins. Sci China Life Sci. 2022;65:280–94. 34387838 10.1007/s11427-021-1964-4 PMC8362648

[22] Walls AC Park Y Tortorici MA. Structure, function, and antigenicity of the SARS-CoV-2 spike glycoprotein. Cell. 2020;183:1735. 33306958 10.1016/j.cell.2020.11.032 PMC7833104

[23] Jackson CB Farzan M Chen B. Mechanisms of SARS-CoV-2 entry into cells. Nat Rev Mol Cell Biol. 2022;23:3–20. 34611326 10.1038/s41580-021-00418-x PMC8491763

[24] Ravi V Saxena S Panda PS. Basic virology of SARS-CoV-2. Indian J Med Microbiol. 2022;40:182–6. 35300895 10.1016/j.ijmmb.2022.02.005 PMC8919811

[25] Santos-López G Cortés-Hernández P Vallejo-Ruiz V. SARS-CoV-2: basic concepts, origin and treatment advances. Gac Med Mex. 2023;157:84–9. 10.24875/GMM.M21000524 34125824

[26] Cao Y Yang R Lee I. Characterization of theSARS-CoV-2 E protein: sequence, structure, viroporin, and inhibitors. Protein Sci. 2021;30:1114–30. 33813796 10.1002/pro.4075 PMC8138525

[27] Liu DX Fung TS Chong KK. Accessory proteins of SARS-CoV and other coronaviruses. Antiviral Res. 2014;109:97–109. 24995382 10.1016/j.antiviral.2014.06.013 PMC7113789

[28] Perdikari TM Murthy AC Ryan VH. SARS-CoV-2 nucleocapsid protein phase-separates with RNA and with human hnRNPs. EMBO J. 2020;39:e106478. 33200826 10.15252/embj.2020106478 PMC7737613

[29] Dang M Song J. CTD of SARS-CoV-2 N protein is a cryptic domain for binding ATP and nucleic acid that interplay in modulating phase separation. Protein Sci. 2022;31:345–56. 34734665 10.1002/pro.4221 PMC8661809

[30] Peng Y Du N Lei Y. Structures of the SARS-CoV-2 nucleocapsid and their perspectives for drug design. EMBO J. 2020;39:e105938. 32914439 10.15252/embj.2020105938 PMC7560215

[31] Burbelo PD Riedo FX Morishima C. Detection of nucleocapsid antibody to SARS-CoV-2 is more sensitive than antibody to spike protein in COVID-19 patients. medRxiv. 2020;222:206–13.

[32] Ni L Ye F Cheng M. Detection of SARS-CoV-2-specific humoral and cellular immunity in COVID-19 convalescent individuals. Immunity. 2020;52:971–7.e3. 32413330 10.1016/j.immuni.2020.04.023 PMC7196424

[33] Xiang F Wang X He X. Antibody detection and dynamic characteristics in patients with coronavirus disease 2019. Clin Infect Dis. 2020;71:1930–4. 32306047 10.1093/cid/ciaa461 PMC7188146

[34] Cascarina SM Ross ED. A proposed role for the SARS-CoV-2 nucleocapsid protein in the formation and regulation of biomolecular condensates. FASEB J. 2020;34:9832–42. 32562316 10.1096/fj.202001351 PMC7323129

[35] Chen H Cui Y Han X. Liquid–liquid phase separation by SARS-CoV-2 nucleocapsid protein and RNA. Cell Res. 2020;30:1143–5. 32901111 10.1038/s41422-020-00408-2 PMC7477871

[36] Zhao M Yu Y Sun L. GCG inhibits SARS-CoV-2 replication by disrupting the liquid phase condensation of its nucleocapsid protein. Nat Commun. 2021;12:2114. 33837182 10.1038/s41467-021-22297-8 PMC8035206

[37] Cascarina SM Ross ED. Phase separation by the SARS-CoV-2 nucleocapsid protein: consensus and open questions. J Biol Chem. 2022;298:101677. 35131265 10.1016/j.jbc.2022.101677 PMC8813722

[38] Chau B Chen V Cochrane AW. Liquid-liquid phase separation of nucleocapsid proteins during SARS-CoV-2 and HIV-1 replication. Cell Rep. 2023;42:111968. 36640305 10.1016/j.celrep.2022.111968 PMC9790868

[39] Wang S Dai T Qin Z. Targeting liquid–liquid phase separation of SARS-CoV-2 nucleocapsid protein promotes innate antiviral immunity by elevating MAVS activity. Nat Cell Biol. 2021;23:718–32. 34239064 10.1038/s41556-021-00710-0

[40] Iserman C Roden CA Boerneke MA. Genomic RNA elements drive phase separation of the SARS-CoV-2 nucleocapsid. Mol Cell. 2020;80:1078–91.e6. 33290746 10.1016/j.molcel.2020.11.041 PMC7691212

[41] Savastano A Ibáñez De Opakua A Rankovic M. Nucleocapsid protein of SARS-CoV-2 phase separates into RNA-rich polymerase-containing condensates. Nat Commun. 2020;11:6041. 33247108 10.1038/s41467-020-19843-1 PMC7699647

[42] Dang M Li T Song J. ATP and nucleic acids competitively modulate LLPS of the SARS-CoV2 nucleocapsid protein. Commun Biol. 2023;6:80. 36681763 10.1038/s42003-023-04480-3 PMC9862227

